# Fabrication of a 3D bioprinting model for posterior capsule opacification using GelMA and PLMA hydrogel-coated resin

**DOI:** 10.1093/rb/rbae020

**Published:** 2024-03-01

**Authors:** Xin Liu, Jiale Li, Shuyu Liu, Yan Long, Ching Kang, Chen Zhao, Ling Wei, Shaoqi Huang, Yi Luo, Bo Dai, Xiangjia Zhu

**Affiliations:** Cataract and Lens Refractive Surgery Group, Department of Ophthalmology, Eye & ENT Hospital of Fudan University, Shanghai 200031, People’s Republic of China; NHC Key Laboratory of Myopia, Key Laboratory of Myopia, Chinese Academy of Medical Sciences, Fudan University, Shanghai 200031, People’s Republic of China; Shanghai Key Laboratory of Visual Impairment and Restoration, Shanghai 200031, People’s Republic of China; Engineering Research Center of Optical Instrument and System, the Ministry of Education, Shanghai Key Laboratory of Modern Optical System, University of Shanghai for Science and Technology, Shanghai 200093, China; Cataract and Lens Refractive Surgery Group, Department of Ophthalmology, Eye & ENT Hospital of Fudan University, Shanghai 200031, People’s Republic of China; NHC Key Laboratory of Myopia, Key Laboratory of Myopia, Chinese Academy of Medical Sciences, Fudan University, Shanghai 200031, People’s Republic of China; Shanghai Key Laboratory of Visual Impairment and Restoration, Shanghai 200031, People’s Republic of China; Engineering Research Center of Optical Instrument and System, the Ministry of Education, Shanghai Key Laboratory of Modern Optical System, University of Shanghai for Science and Technology, Shanghai 200093, China; Cataract and Lens Refractive Surgery Group, Department of Ophthalmology, Eye & ENT Hospital of Fudan University, Shanghai 200031, People’s Republic of China; NHC Key Laboratory of Myopia, Key Laboratory of Myopia, Chinese Academy of Medical Sciences, Fudan University, Shanghai 200031, People’s Republic of China; Shanghai Key Laboratory of Visual Impairment and Restoration, Shanghai 200031, People’s Republic of China; Cataract and Lens Refractive Surgery Group, Department of Ophthalmology, Eye & ENT Hospital of Fudan University, Shanghai 200031, People’s Republic of China; NHC Key Laboratory of Myopia, Key Laboratory of Myopia, Chinese Academy of Medical Sciences, Fudan University, Shanghai 200031, People’s Republic of China; Shanghai Key Laboratory of Visual Impairment and Restoration, Shanghai 200031, People’s Republic of China; Cataract and Lens Refractive Surgery Group, Department of Ophthalmology, Eye & ENT Hospital of Fudan University, Shanghai 200031, People’s Republic of China; NHC Key Laboratory of Myopia, Key Laboratory of Myopia, Chinese Academy of Medical Sciences, Fudan University, Shanghai 200031, People’s Republic of China; Shanghai Key Laboratory of Visual Impairment and Restoration, Shanghai 200031, People’s Republic of China; Engineering Research Center of Optical Instrument and System, the Ministry of Education, Shanghai Key Laboratory of Modern Optical System, University of Shanghai for Science and Technology, Shanghai 200093, China; Cataract and Lens Refractive Surgery Group, Department of Ophthalmology, Eye & ENT Hospital of Fudan University, Shanghai 200031, People’s Republic of China; NHC Key Laboratory of Myopia, Key Laboratory of Myopia, Chinese Academy of Medical Sciences, Fudan University, Shanghai 200031, People’s Republic of China; Shanghai Key Laboratory of Visual Impairment and Restoration, Shanghai 200031, People’s Republic of China; Engineering Research Center of Optical Instrument and System, the Ministry of Education, Shanghai Key Laboratory of Modern Optical System, University of Shanghai for Science and Technology, Shanghai 200093, China; Cataract and Lens Refractive Surgery Group, Department of Ophthalmology, Eye & ENT Hospital of Fudan University, Shanghai 200031, People’s Republic of China; NHC Key Laboratory of Myopia, Key Laboratory of Myopia, Chinese Academy of Medical Sciences, Fudan University, Shanghai 200031, People’s Republic of China; Shanghai Key Laboratory of Visual Impairment and Restoration, Shanghai 200031, People’s Republic of China

**Keywords:** posterior capsule opacification (PCO), human lens epithelial cells (HLECs), 3D bioprinting, hydrogel, resin

## Abstract

Posterior capsule opacification (PCO) remains the predominant complication following cataract surgery, significantly impairing visual function restoration. In this study, we developed a PCO model that closely mimics the anatomical structure of the crystalline lens capsule post-surgery. The model incorporated a threaded structure for accurate positioning and observation, allowing for opening and closing. Utilizing 3D printing technology, a stable external support system was created using resin material consisting of a rigid, hollow base and cover. To replicate the lens capsule structure, a thin hydrogel coating was applied to the resin scaffold. The biocompatibility and impact on cellular functionality of various hydrogel compositions were assessed through an array of staining techniques, including calcein-AM/PI staining, rhodamine staining, BODIPY-C11 staining and EdU staining in conjunction with transwell assays. Additionally, the PCO model was utilized to investigate the effects of eight drugs with anti-inflammatory and anti-proliferative properties, including 5-aminoimidazole-4-carboxamide ribonucleotide (AICAR), THZ1, sorbinil, 4-octyl itaconate (4-OI), xanthohumol, zebularine, rapamycin and caffeic acid phenethyl ester, on human lens epithelial cells (HLECs). Confocal microscopy facilitated comprehensive imaging of the PCO model. The results demonstrated that the GelMA 60 5% + PLMA 2% composite hydrogel exhibited superior biocompatibility and minimal lipid peroxidation levels among the tested hydrogels. Moreover, compared to using hydrogel as the material for 3D printing the entire model, applying surface hydrogel spin coating with parameters of 2000 rpm × 2 on the resin-based 3D printed base yielded a more uniform cell distribution and reduced apoptosis. Furthermore, rapamycin, 4-OI and AICAR demonstrated potent antiproliferative effects in the drug intervention study. Confocal microscopy imaging revealed a uniform distribution of HLECs along the anatomical structure of the crystalline lens capsule within the PCO model, showcasing robust cell viability and regular morphology. In conclusion, the PCO model provides a valuable experimental platform for studying PCO pathogenesis and exploring potential therapeutic interventions.

## Graphical abstract

**Figure:**
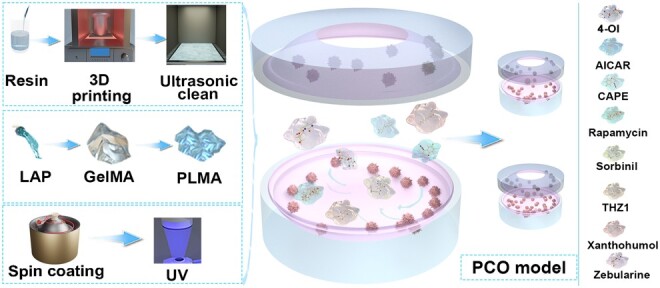

## Introduction

Cataracts, the leading cause of treatable blindness worldwide, necessitate phacoemulsification surgery as the primary treatment [1, 2]. However, the occurrence of posterior capsule opacification (PCO) remains a significant complication following this procedure [3]. Although neodymium: yttrium-aluminum-garnet (Nd: YAG) laser posterior capsulotomy serves as the standard treatment, it has associated risks, such as intraocular lens (IOL) damage or dislocation, increased inflammation and retinal detachment [4]. In the era of refractive cataract surgery with advanced functional IOL implantation, even mild PCO can severely impact visual quality, leading to patient dissatisfaction. Moreover, complex cataract surgeries in cases such as highly myopic or postvitrectomy complicated cataracts are susceptible to complications such as capsular bag contraction syndrome.

Currently, research on PCO prevention focuses on reducing residual lens epithelial cells or regulating their cellular behavior [5]. This intervention strategy is closely related to the biocompatibility of IOLs. Therefore, scientists have conducted extensive studies using strategies such as IOL surface modification or IOL bulk materials design for PCO prevention [3]. Strategies for IOL surface improvement include ultraviolet (UV)/ozone treatment, layer-by-layer self-assembly methods, drug loading and coating modification [5–7]. Previous IOL surface coating modifications have led to the development of anti-inflammatory IOLs, micro-patterned IOLs, enhanced adhesion IOLs, photo-responsive IOLs and drug-loading IOLs [3]. The latest research indicates that nanoparticles loaded with drugs can slow down the proliferation of HLECs, and nanoparticles-modified IOL could significantly inhibit the incidence of PCO, offering broad application prospects [8]. The anti-proliferative drug doxorubicin (DOX) was initially used in nano-particle-loaded IOLs for PCO prevention, leading to the development of a polysaccharide multilayer modified IOL with DOX-loaded chitosan nanoparticles, demonstrating anti-proliferative and anti-adhesive properties [9]. This led to further development of NLCs encapsulating genistein, a potential TKI, which can inhibit the proliferation, migration, and transformation of HLECs [10]. The anti-metabolic drug 5-FU was also encapsulated into chitosan nanoparticles to improve IOL through sustained release systems [11]. Recent studies have revealed that the non-steroidal anti-inflammatory drug (NSAID), bromfenac, can hinder TGF-β2-induced cell migration and the EMT of LECs through ERK/GSK-3β/Snail signaling. Building on this discovery, researchers further developed an innovative nano-particle-enhanced IOL-sustained release system called the drug-eluting IOL, utilizing poly(lactic-co-glycolic acid) with prolonged bromfenac release capability to prevent PCO development [12]. These strategies all show promise for preventing the occurrence and development of PCO and have good prospects for application.

In order to further improve and develop strategies for the prevention and treatment of PCO, it is crucial to conduct in-depth research into the mechanisms underlying the development of PCO. Therefore, a comprehensive understanding of the fundamental mechanisms contributing to PCO development is essential [13]. The mechanisms underlying the development of PCO are not yet fully understood. Several factors have been proposed to contribute to its occurrence, including inflammatory responses, capsular bag contraction, age, and the design and material properties of IOLs, which trigger the proliferation, migration and transdifferentiation of human lens epithelial cells (HLECs) [14–16]. Understanding these mechanisms can contribute to the development of preventive strategies and interventions aimed at minimizing the occurrence of PCO and enhancing visual outcomes following cataract surgery.

To study the potential mechanisms of PCO, several models could be adopted, including *in vitro* cell or tissue cultivation, *in vitro* capsular bag models and animal models [17–20]. Each model has its own advantages and weaknesses. The lens capsule is an extremely delicate and pliable structure that provides a framework for the proliferation of HLECs. However, the fragility of the lens capsule presents challenges in constructing models that offer adequate support. Consequently, several support methods have been developed to overcome this hurdle. These methods include needle insertion, capsule tension rings, silicone tubes and 3D-printed support structures [19, 21, 22]. Furthermore, the development of an anterior chamber model that replicates the PCO microenvironment with the inclusion of various growth factors in the culture media has been established [19]. These support methods, however, may sometimes lead to distortions or unintended contact with the lens capsule, resulting in inconsistent and unreliable outcomes. These artifacts can introduce confounding factors and interfere with the accuracy and reproducibility of experimental results.

To enhance the structural support and stability in experimental PCO models, we develop a novel capsular bag model. This involves fabricating a 3D bioprinted PCO model with a hydrogel coating composed of gelatin methacryloyl (GelMA) and methacrylamide-modified ε-poly-l-lysine (ε-PLMA) on resin. By accurately replicating the structural and parameter characteristics of the human lens, the proposed PCO model provides a platform for investigating the development and progression of PCO. The utilization of a combined hydrogel material for internal coating creates an optimal microenvironment and scaffold for HLEC growth within the model. This not only supports the viability and functionality of lens epithelial cells but also enables the evaluation of various cellular processes involved in PCO pathogenesis. Moreover, the design of an open lid allows for the separation and observation of different stages or treatments, facilitating a detailed assessment of drug effects. This feature enhances the model’s suitability for drug research, enabling the screening and identification of potential therapeutic candidates for PCO treatment. Overall, this innovative technique aims to provide a more reliable, reproducible and controlled environment for the growth and examination of HLECs, thereby improving the accuracy and consistency of research conducted in the field of PCO.

## Materials and methods

### Materials

The HLEC line (SRA01/04) was obtained from the Cell Resource Center, Shanghai Institutes for Biological Sciences, Chinese Academy of Sciences. DAPI, calcein-AM and propidium iodide (PI) were provided by Shanghai Beyotime Biotechnology Co., Ltd (Shanghai, China). The CCK-8 kit was provided by Dojindo Laboratories (Kumamoto, Japan). Phosphate-buffered saline (PBS) was provided by HyClone. Penicillin–streptomycin, fetal bovine serum (FBS) and Dulbecco’s modified Eagle’s medium (DMEM) were provided by Gibco (USA). Clear resin was provided by Raise3D (Shanghai, China). GelMA and methacrylamide-modified ε-poly-l-lysine (ε-PLMA) hydrogels were provided by EFL Co., Ltd (Suzhou, China). The CellTracker™ Blue CMAC fluorescent probe and the BODIPY 581/591 C11 kit (D3861) were provided by Thermo Fisher Scientific (USA). Anti-fade fluorescence mounting medium—aqueous fluoroshield (ab104135) was provided by Abcam (USA). The 5-ethynyl-20-deoxyuridine (EdU) Cell Proliferation Apollo567 Kit was provided by RiboBio (Guangzhou, China). The PrimeScript™ RT Reagent Kit and SYBR Green detection kit were provided by Takara Bio, Inc. (Japan). Pharmacological agents, including 5-aminoimidazole-4-carboxamide ribonucleotide (AICAR), THZ1, sorbinil, 4-octyl itaconate (4-OI) and xanthohumol, were provided by MedChemExpress (MCE, USA). Zebularine, rapamycin and caffeic acid phenethyl ester (CAPE) were provided by Aladdin Biochemical Technology Co., Ltd (Shanghai, China).

### Model design

The PCO model was designed based on anatomical parameters of the human lens, with a horizontal diameter of 11.665 mm, vertical diameter of 4.023 mm and posterior curvature radius of 10 mm. The anterior surface simulated a circular opening of 5.5 mm in diameter that was created by continuous circular capsulorhexis during cataract surgery. A reserved groove of 1 mm was thoughtfully included at the equator of the PCO model to accommodate the inoculated cells.

**Figure 1.**
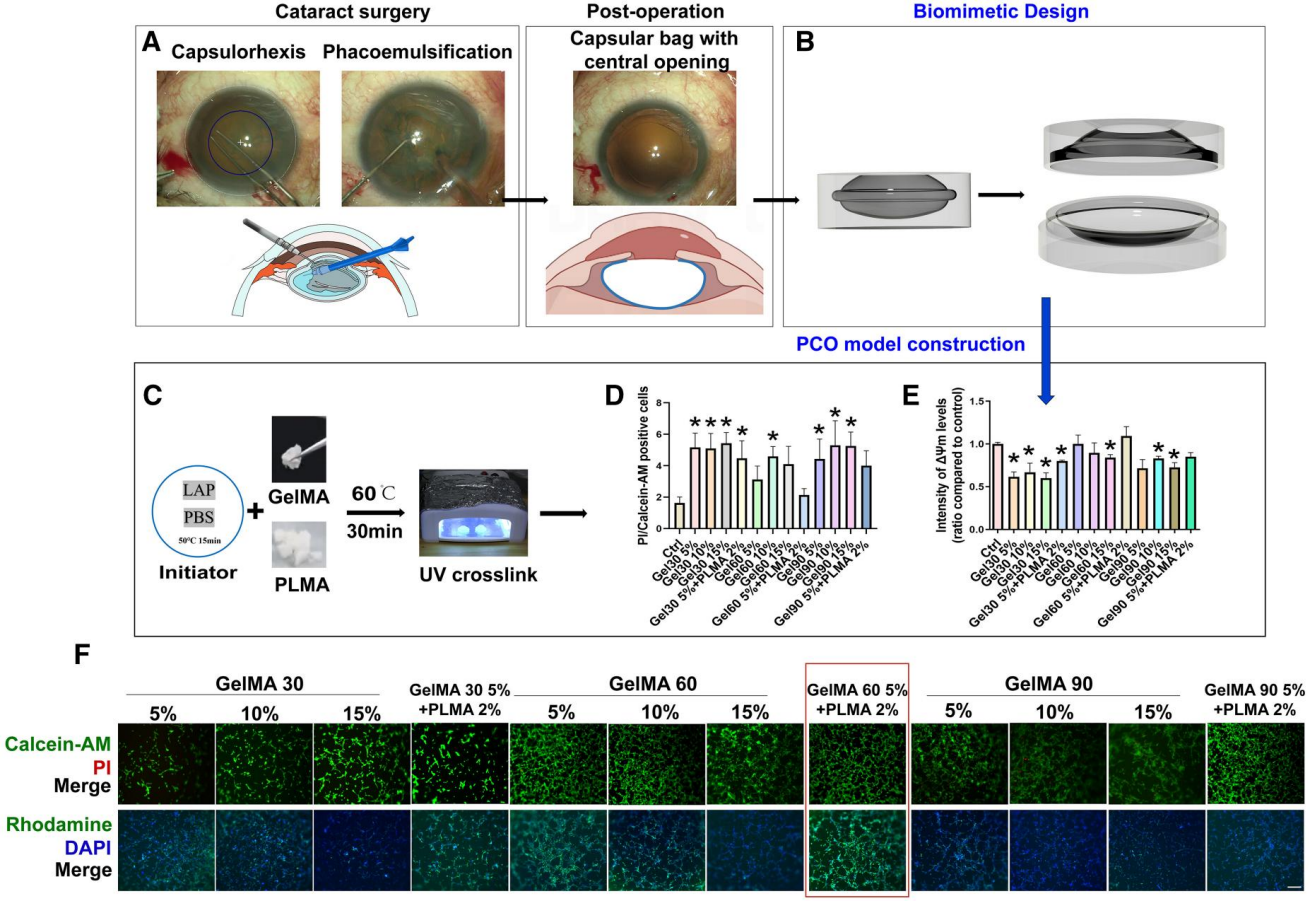
Design and construction of the PCO model. (**A**) Intraoperative images of capsulorhexis and phacoemulsification as well as postoperative images and cross-sectional illustrations of the procedures. (**B**) Design of the openable PCO model with upper and lower parts based on postoperative conditions. (**C**) Workflow for hydrogel preparation. (**D–F**) HLECs were cultured in different concentrations and strengths of hydrogels, including GelMA with 30%, 60% and 90% amino substitution degrees at concentrations of 5%, 10% and 15%, with or without PLMA 2%. (D) The ratio of PI-positive cells (apoptotic cells) to calcein-AM-positive cells (live cells) among different groups. (E) Intensity of mitochondrial membrane potential compared to the control group. (F) Apoptotic cells were analyzed using calcein-AM and PI double staining, where green represents live cells and red represents dead or dying cells. Mitochondrial membrane potentials were detected using rhodamine 123 and DAPI double staining in different groups. Scale bar= 50 μm. Statistical significance was denoted as *, *P* < 0.05 (*n* = 3).

To accurately replicate the post-cataract surgery shape, the entire model took the form of an external cylindrical base with a hollow structure, without the central lens. To facilitate observation and intervention, the PCO model was designed with an openable structure. The upper and lower parts of the model were connected by threads, and a groove was specifically incorporated to accommodate cell inoculation. After the upper and lower parts were opened and cells were inoculated, they could be securely fastened along the curve of the model. The entire model was then filled with a culture medium to support cell growth. After performing the desired interventions, the model could be opened for staining and observation (Figure 1A and B).

For the outer base of the model, a resin material was used for 3D printing, creating a central hollow. The surface of the hollow was then coated with an ultrathin layer of hydrogel by spin coating at different parameters. This hydrogel replicated the structure of the lens capsule, providing a scaffold for optimal cell culture conditions.

### Preparation of hydrogels

For the preparation of the 0.25% (w/v) initiator standard solution, 20 ml of PBS was added to a brown vial containing 0.05 g of LAP. The mixture was gently agitated and heated in a water bath at 40–50°C for 15 min with intermittent stirring. GelMA with 30%, 60% and 90% amino substitution degrees was used in this study (called GM30, GM60, and GM90). Under equivalent conditions, higher substitution degrees result in stronger hydrogels after curing. Methacrylated polylysine (PLMA), a modified form of polylysine with double bonds, exhibited broad-spectrum antibacterial and antifungal activities. The gelation of PLMA was achieved through crosslinking under the action of a photoinitiator using UV or visible light. To prepare the hydrogel solution, the desired amount of GelMA or PLMA was weighed and placed into a centrifuge tube (2% concentration corresponds to 20 mg/ml, 5–50 mg/ml, 10–100 mg/ml and 15–150 mg/ml). The mixed hydrogel solution was prepared by dissolving PLMA completely at room temperature using an initiator standard solution, followed by the addition of GelMA. The mixture or the pure GelMA hydrogel solution was heated in a light-shielded water bath at 60–70°C for 20–30 min with periodic agitation. Finally, the GelMA solution was immediately sterilized using a 0.22-μm sterile syringe filter to prevent gelation at low temperatures (Figure 1C). Afterward, the hydrogel solution was either injected into a well plate or spin-coated onto a PCO model. The gelation process was induced by irradiating the samples with a 405 nm light source for a period of 30–60 s.

### 3D printing of the PCO model

The designed parameters were input into the Form 3+ SLA 3D printer using Clear resin as the printing material known for its optical transparency, smooth surface finish and high resolution. The printing process took approximately 2 h and 18 min. After printing, the model was subjected to ultrasonic cleaning for 3 min and underwent UV curing for 15 min.

**Figure 2.**
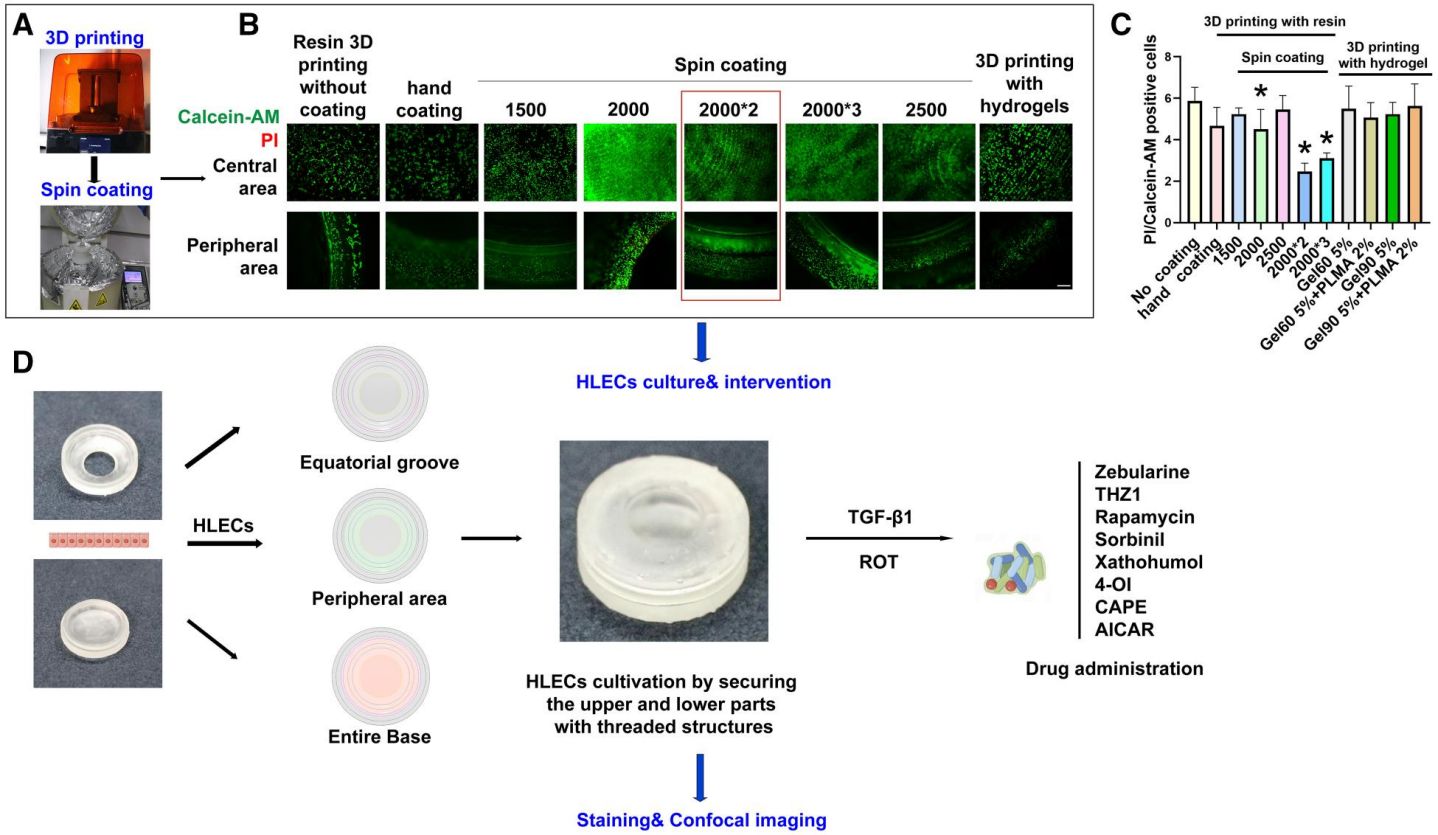
Schematic diagram of PCO model construction, intervention and analysis. (**A**) Workflow for resin 3D printing of the base and cover and surface spin coating. (**B)** Calcein-AM and PI double staining for live and dead cell staining, respectively. Calcein-AM positive represents live cells, while PI positive indicates dead or dying cells. The central portion and the peripheral region of the PCO model were visualized. Scale bar = 50 μm. A comparison of different surface coating methods on the resin 3D-printed PCO model and a comparison of using hydrogel as the material for 3D printing the PCO model. (**C**) The ratio of PI-positive cells (apoptotic cells) to calcein-AM-positive cells (live cells) among the different groups. Scale bars: 50 μm. Statistical significance was denoted as *, *P* < 0.05 (*n* = 3). (**D**) HLECs can be seeded and adhered to in the PCO model using three different methods: seeding in the groove at the equator of the PCO model, adhering to the periphery after confinement by pillars, or seeding cells on the entire base model. The upper and lower parts are then closed for cultivation. Subsequently, drug interventions are conducted under pathological conditions, followed by staining and imaging using confocal microscopy.

The model was securely fixed to a glass slide and placed in a Laurell spin coater. The GelMA hydrogel, prepared as described previously, was heated to 60°C and then injected into the central area of the PCO model, with a volume of 200 μl. The spin-coating parameters were set at 2000 rpm for two rounds, followed by UV curing for 1 min. Afterward, the model coated with hydrogels was soaked in 75% alcohol for 1 h to ensure sterilization before being used for cell culture **(**Figure 2A**)**. A video demonstrating the entire model preparation process is provided (see Supplementary Video 1).

The video demonstrates the process of PCO model design and construction. It shows the design of the PCO model, inputting the parameters required for 3D printing, and utilizing resin material for the printing process. The video also showcases the steps of ultrasonic cleaning, UV curing, hydrogel spin coating and UV cross-linking. This comprehensive process is essential for creating a well-designed and functional PCO model.

As a comparison, EFL-BP-8601 Pro, a photopolymer-based 3D printer from EFL Company (Suzhou, China), was used to print the external hard base of the entire PCO model using a GelMA hydrogel. The printing parameters were set as follows: light intensity density of 10 mW/cm^2^, exposure time of 10 s, layer height of 100 μm, peel distance of 3 mm, peel speed of 25 mm/min and lift speed of 100 mm/min. After printing, the model was soaked in 75% alcohol for 1 h to ensure sterilization before being used for cell culture.

### Cell culture

The HLEC-SRA01/04 cells were cultured in DMEM (high glucose) (Gibco, USA) supplemented with 10% FBS (Gibco, USA), 100 mg/ml streptomycin and 100 units/ml penicillin. The cells were maintained at 37°C in a humidified atmosphere with 5% CO_2_, following a previously described protocol [23].

To prepare the cell suspension, the cells were counted and adjusted to a density of 1 × 10^6^ cells/ml. Subsequently, the cells were seeded onto the surface of the hydrogel or PCO model. The culture medium was replaced every other day to maintain optimal conditions for cell growth.

### Comparison and selection of optimal hydrogel mixture

In order to compare different hydrogel mixtures and select the most suitable formulation for coating the surface of the PCO model, we utilized calcium-AM/PI double staining, live cell tracking and Rhodamine 123 staining to evaluate the biocompatibility and cell viability of various formulations. By comparing the results of these formulations, the optimal hydrogel mixture for coating the PCO model surface can be selected to ensure optimal cell growth and function. Calcein-AM and PI double staining were performed to distinguish live and dead cells as described previously [24]. The cells were seeded into the precoated hydrogel in a 24-well plate, or the PCO model was placed in a 24-well plate, and HLECs were subsequently seeded onto the equatorial groove or base of the PCO model. After culturing for 48 h, the culture medium was removed, and the cells were washed three times with PBS. A PI and calcein-AM dilution was prepared by adding 1 μl of PI and 1 μl of calcein-AM to 1 ml of DMEM (final concentration of 10 μM). Each well was then incubated with 200 μl of the PI and calcein-AM dilution at 37°C for 30 min. After three washes with PBS, two drops of anti-Fade fluorescence mounting medium were added to prevent fluorescence quenching. The 24-well plate was placed under an inverted fluorescence microscope, and live cells were observed using a 490-nm excitation filter (green), while dead cells were observed using a 545 nm excitation filter (red). Images were captured, cell counts were recorded and further analysis was performed.

The CellTracker™ Blue CMAC fluorescent probe was used for live cell staining and dynamic observation from day 1 to day 7. The staining protocol for adherent cells involved the following steps: removal of culture media, gentle addition of prewarmed CellTracker™ Working Solution, incubation for 15–45 min under growth conditions suitable for the specific cell type, removal of the working solution, addition of desired culture media and imaging of stained cells using appropriate excitation (353 nm) and emission (466 nm) filters for the CellTracker™ probe.

Rhodamine 123, a cationic fluorescent dye, was used to assess mitochondrial membrane potential. After a 48-h incubation period in precoated hydrogel within a 24-well plate, the cells were washed three times with PBS. A final concentration of 2 μM rhodamine 123 was prepared by dilution and added to each well, followed by incubation at 37°C for 30 min. Subsequently, the cells were rinsed with PBS, and DAPI was diluted to a final concentration of 100 μg/ml and added to each well. The plate was then incubated at 37°C for 5 min. An inverted fluorescence microscope was employed to visualize the green fluorescence intensity, reflecting the strength of the mitochondrial membrane potential, using an excitation wavelength of 490 nm. Additionally, the cell nuclei were stained blue and observed using an excitation wavelength of 450 nm. Images were captured, and cell counts were recorded and analyzed.

### Evaluation of oxidative stress levels

The assessment of lipid peroxidation levels was performed using the BODIPY 581/591 C11 kit. Cells were either seeded into a precoated hydrogel in a 24-well plate, or HLECs were seeded onto the equatorial groove or base of the PCO model placed in a 24-well plate. After 48 h of culture, the culture medium was removed, and the cells were washed three times with PBS. Subsequently, the cells were incubated with the BODIPY-C11 probe at a final concentration of 5 µM in the medium for 1 h at 37°C. Following three additional washes with PBS, anti-fade fluorescence mounting medium was added to prevent fluorescence quenching. The 24-well plate was then placed under an inverted fluorescence microscope. Upon oxidation–reduction reactions, the emission wavelength shifted from 591 nm (reduced form, orange fluorescence) to 510 nm (oxidized form, green fluorescence). The fluorescence density of the oxidized form reflected the extent of oxidative stress and was compared to that of the control group.

### Drug administration

To explore the impact of the PCO model on drug screening, we selected eight pharmacological agents with antiapoptotic, anti-inflammatory and antioxidant properties. These compounds were employed to intervene in the cell function of HLECs, aiming to observe their effects on cell proliferation, migration and apoptosis.

Zebularine, a DNA methyltransferase inhibitor, exhibited a dose-dependent inhibition of HLEC proliferation by downregulating PI3K/Akt and MAPK signaling [25, 26]. AICAR, an activator of AMP-activated protein kinase (AMPK), operates as a permeable adenosine monophosphate analog. It plays a vital role in regulating energy metabolism, modulating glucose and lipid metabolism, and inhibiting the production of proinflammatory cytokines and iNOS. Additionally, AICAR functions as an autophagy, YAP and mitophagy inhibitor [27]. THZ1, a selective covalent CDK7 inhibitor, has shown potential as a novel treatment for PCO by suppressing TGFβ2-mediated epithelial-mesenchymal transition (EMT) in HLECs through the Notch and TGFβ/Smad signaling pathways [28]. Rapamycin (Sirolimus), a specific mTOR inhibitor, effectively suppresses the proliferation and migration of HLECs mediated by the AKT/mTOR signaling pathway in response to certain growth factors [29]. Sorbinil, an aldose reductase inhibitor, contributes to PCO prevention by blocking the MAPK signaling pathway, thereby inhibiting LEC proliferation and EMT [30, 31]. 4-OI, an endogenous salicylate derivative with cell membrane permeability, exerts its anti-inflammatory effects by upregulating nuclear factor erythroid 2-related factor 2 (Nrf2) and downregulating p38 signaling [32]. xanthohumol, a flavonoid compound, exhibits remarkable antioxidant, anti-inflammatory, antibacterial, antifungal and antitumor properties [33]. CAPE, the main active component of propolis and a flavonoid, possesses diverse pharmacological potentials, such as anticancer, anti-inflammatory, antioxidant, antibacterial and antifungal effects, effectively protecting multiple organs [34].

The interventions of these drugs in the PCO model and cell culture were as follows: Zebularine (10 μM), THZ1 (400 nM), Rapamycin (200 nM), Sorbinil (100 nM), xanthohumol (20 μM), 4-OI (100 μM), CAPE (20 μM) and AICAR (50 μM).

### Cell migration evaluation

Cell proliferation was assessed using the 5-ethynyl-20-deoxyuridine (EdU) Cell Proliferation Apollo567 Kit. EdU is an analog of thymidine (T) that is incorporated into actively synthesized DNA during cell proliferation. After 3 days of drug treatment, HLECs seeded in the PCO model were incubated with 50 μM EdU for 3 h. Subsequently, each well was incubated with cell fixation solution at room temperature for 30 min. Neutralization was achieved by adding 2 mg/ml glycine, and cell permeability was enhanced using a permeabilization buffer containing 0.5% Triton X-100. For labeling proliferating cells, each model received 1× Apollo^®^ staining reaction solution and was incubated on a rocking shaker at room temperature in the dark for 30 min. Cell nuclei were counterstained with Hoechst 33342. Immunoﬂuorescence was observed using a fluorescence microscope. Cell proliferation was detected using an excitation wavelength of 545 nm, followed by nuclear observation using an excitation wavelength of 450 nm.

The Transwell assay was employed to evaluate the migratory capacity of HLECs. HLECs in the logarithmic growth phase were seeded in a PCO model. Following drug intervention, the cells were trypsinized, terminated and centrifuged. The resulting cell suspension with a density of 1 × 10^6^ cells/ml was prepared using serum-free DMEM. Subsequently, 100 μl of the cell suspension was added to the central upper chamber of an 8 μm Transwell hanging chamber. The lower chamber was filled with DMEM (high glucose) supplemented with 15% FBS to stimulate cell migration toward it. The chambers were then incubated at 37°C in a cell culture incubator for 48 h. After removing the chambers, nonmigrated cells on the upper surface of the membrane were gently wiped off. The chambers were washed, fixed with formaldehyde for 15 min and stained with 0.1% crystal violet staining solution for 20 min. The chambers were inverted and placed in a 50°C oven for 1 h to ensure complete drying. Finally, the number of cells that migrated through the membrane was quantified under a microscope.

### Confocal microscopy

For live-cell imaging, HLECs were seeded onto the PCO model at a density of 1 × 10^6^ cells/ml. Calcein-AM staining was performed to visualize the live cells. The PCO model was placed in a 24-well plate, and HLECs were subsequently seeded onto the equatorial groove, peripheral area or base of the PCO model. Following intervention, the culture medium was removed, and the cells were washed three times with PBS. A calcein-AM dilution (final concentration: 10 μM) was applied to each well, followed by incubation at 37°C for 30 min. After washing, anti-Fade fluorescence mounting medium was added to prevent fluorescence quenching.

Subsequently, the inverted PCO model in the confocal dish was imaged using a ZEISS LSM 900 confocal microscope utilizing a 490-nm excitation filter (green) and 5× magnification. The multidimensional acquisition mode was utilized to capture Z-stack images with a range of 1900 μm and an interval of 50 μm. Tile regions of 14 × 14 (176 tiles, total area: 16228.1 μm^2^) were employed, necessitating approximately 9 h for complete acquisition. To enhance details, further imaging was conducted specifically on the central region of the PCO model base. This involved Z-stack images with a range of 1000 μm and an interval of 35 μm. Tile regions of 8 × 8 (64 tiles) were used, with an acquisition time of approximately 2 h. After drug intervention, images of the core region of the PCO model base were captured. This included Z-stack images with a range of 100 μm and an interval of 8 μm. Tile regions of 3 × 3 (nine tiles) were employed, with an acquisition time of approximately 15 min.

Parameters settings were as follows:

Track 1: Laser wavelength: 488 nm: 0.2%. Channel 1: Excitation wavelength: 494, emission wavelength: 514, detection wavelength: 450–620.

**14 × 14 imaging:** Z-Stack: 55 slices (1.89 mm), Tiles: 177 tiles (2 scenes), Scaling (per pixel): 1.248 μm * 1.248 μm * 35.000 μm, Image Center position: X −5.73 mm, Y 3.81 mm.

**8 × 8 imaging:** Z-Stack: 38 slices (296 μm), Tiles: 50 tiles (2 scenes), scaling (per pixel): 1.248 μm * 1.248 μm * 8.000 μm, Image Center position: X −311.75 μm, Y −270.61 μm.

**3 × 3 imaging:** Z-Stack: 13 slices (96 μm), Tiles: 17 tiles (2 scenes), Scaling (per pixel): 1.248 μm * 1.248 μm * 8.000 μm, Image Center position: X −36.61 mm, Y −3.79 mm.

### Statistical analysis

The acquired images from this experiment were analyzed using ImageJ software. Numerical data were analyzed using GraphPad Prism Version 7.0 (GraphPad Software, Inc., La Jolla, CA, USA) and are presented as the mean ± standard error of the mean (SEM). Statistical significance was assessed using two-tailed Student’s *t* test or one-way ANOVA followed by *post hoc* Bonferroni correction using SPSS 16.0 (SPSS Inc., USA). A *P* value <0.05 was considered to indicate statistical significance.

## Results and discussion

### 3D printing and PCO model construction

This study explores the methodology for constructing an *in vitro* PCO model. Initially, the external design of the model was based on the anatomical structure of the lens and the structure of the lens capsule after cataract surgery (Figure 1A and B). Resin materials were then utilized for 3D printing to create a stable outer layer structure. The entire model was divided into an upper and lower opening structure through a central screw design. This design not only allows precise positioning of HLECs in the central equatorial region but also enables the cultivation and simulation of the PCO microenvironment for drug intervention. Additionally, the upper and lower bases of the model can be separated for staining and photography purposes. To simulate the ultrathin structure of the capsular bag, a combination of hydrogel formulas was compared. GelMA 60 5%+PLMA 2% was selected because it exhibited the best biocompatibility and viability (Figure 1D–F). Optimal surface coating for the PCO model involved a thin gel layer applied through a two-round spin-coating scheme at 2000 rpm, resulting in uniform cell adherence, improved growth status, and minimal lipid peroxidation (Figure 2A–C). After the completion of model construction, UV cross-linking was performed, followed by 1-h sterilization in 75% alcohol for cell culture.

In this study, three different methods were employed to simulate the PCO model. First, HLECs were seeded in the equatorial groove to mimic cell migration in the equatorial region. Second, using a central pillar, central HLECs were restricted and seeded in the periphery of the PCO model to simulate overall cell migration. Finally, HLECs were seeded at the bottom of the entire PCO model to observe overall cell growth. These three methods allow for the application of the PCO model based on research requirements and intervention strategies. After sealing the model, the microenvironment of PCO is simulated, and drug interventions can be performed on HLECs. Once the intervention is completed, the upper and lower covers can be separated again for staining and better observation. Due to the limited imaging range of various fluorescence microscopes, especially confocal microscopes, the reduced height of the base after opening enables better imaging within the range of the microscope (Figure 2D). The model is highly stable and can be reused multiple times. After staining, it can be placed in alcohol for 1 h to restore its sterile state without affecting the morphology and functionality of the gel and model base, greatly enhancing convenience and usability.

To explore the pathological mechanisms and drug intervention strategies for PCO, several *in vivo* and *in vitro* models have been developed by researchers [16]. Animal models, including New Zealand rabbits, rats and mice, are commonly utilized to establish *in vivo* PCO models, offering the advantage of simulating the postoperative process following cataract surgery and allowing for the implantation of IOLs. However, these *in vivo* models differ significantly from humans in terms of the inflammatory response and protein expression profile [6, 35]. Another frequently used PCO model involves the *ex vivo* culture of lens capsules. Researchers utilize donated eyes to construct *ex vivo* models of human lens capsules by isolating the intact crystalline lens-zonule-ciliary body complex from the eyeball and fixing it to different ring systems. The entire complex or lens capsule is then supported and fixed in the ciliary process suspensory region *ex vivo.* The advantage of this type of *ex vivo* model is the direct use of human lens capsules for research, making *ex vivo* cultivation relatively convenient and easy to manipulate. However, it lacks the construction of an *in vivo* microenvironment, and the lens capsule is highly prone to collapse and deformation, thus unable to effectively maintain the anatomical structure of the lens [17, 36, 37]. Wertheimer *et al.* developed a 3D-cultured PCO model. In this model, the lens capsule is first separated *ex vivo* after cataract surgery and placed at the bottom of the model. An IOL is then implanted, and close contact between the IOL and the capsule is achieved using a 1-g steel weight. HLECs were injected around the periphery of this culture device to observe their proliferation and migration. This approach offers the advantage of effectively simulating the microenvironment and combining features of *in vivo* and *ex vivo* models. The outer and lower chambers of this anterior chamber model replicate other ocular structures besides the lens, thereby simulating the interaction between the lens and its surrounding microenvironment [19]. Jaitli *et al.* utilized gelatin material to create a ‘simulated lens capsule’ with a curvature that mimics the bag-like structure collapsing on IOL post-implantation. An advantage of this approach is that it facilitates the 3D culture of HLECs [21]. Hiramatsu *et al.* developed a novel human immortalized crystalline lens epithelial cell line (iHLEC-NY1s) and effectively generated a 3D cellular model (3D-iHLEC-NY1s) that resembled the tissue structure of Soemmering’s ring using static and rotation-floating methods. The model expressed proteins such as αA-crystalline, βB2-crystalline and vimentin, indicating that its protein composition closely resembles that of the human crystalline lens [38]. However, breakthroughs are still needed in key areas, including three-dimensional dynamic culture, biomimetic bag-like support and simulation and intervention of the microenvironment, to further improve the construction method of PCO models, making it more convenient, efficient and accurate [13, 16].

In this study, our PCO model was constructed to mimic the biomimetic appearance of the human lens capsule structure. We employed resin-based 3D printing technology to create a stable outer base providing effective support. Additionally, a thin hydrogel coating, resembling the capsule structure, was applied to the surface to establish an optimal growth microenvironment. The open-close design facilitates precise positioning, intervention and staining observation. This model aims to simulate key factors involved in the occurrence and development of PCO for effective interventions in a more convenient manner.

### Selection of the optimal surface coating strategies

The selection of the optimal hydrogel mixture as the ultra-thin coating for the PCO model was determined by assessing cell viability and biocompatibility. To evaluate the cell viability and biocompatibility of HLECs within GelMA hydrogels under various concentration and strength conditions, this study utilized GelMA with 30%, 60% and 90% amino substitution degrees (GM30, GM60 and GM90) at concentrations of 5%, 10% and 15%. The hydrogels were cast into wells of a 24-well plate and seeded with HLECs for cultivation. Calcein-AM/PI staining was employed to observe the number of live and dead cells. The results demonstrated that compared to the control group, the GM30 hydrogel at all three concentrations exhibited collapsing of the gel scaffold, with significantly fewer live cells stained by calcein-AM and a higher number of dead cells stained by PI, compared to the GM60 and GM90 hydrogels. This indicates poorer growth conditions for HLECs within the GM30 hydrogel, which failed to provide adequate support for cell growth. In contrast, the numbers of live cells in the GM60 and GM90 hydrogels at the three concentrations were similar without any statistically significant differences. However, the number of dead cells was significantly higher in the GM90 hydrogel at all three concentrations than in the GM60 hydrogel. Notably, the GelMA 60 5%+PLMA 2% group exhibited the lowest number of dead cells and the lowest ratio of dead cells to live cells among all gel concentrations and strengths (Figure 1D and F).

**Figure 3.**
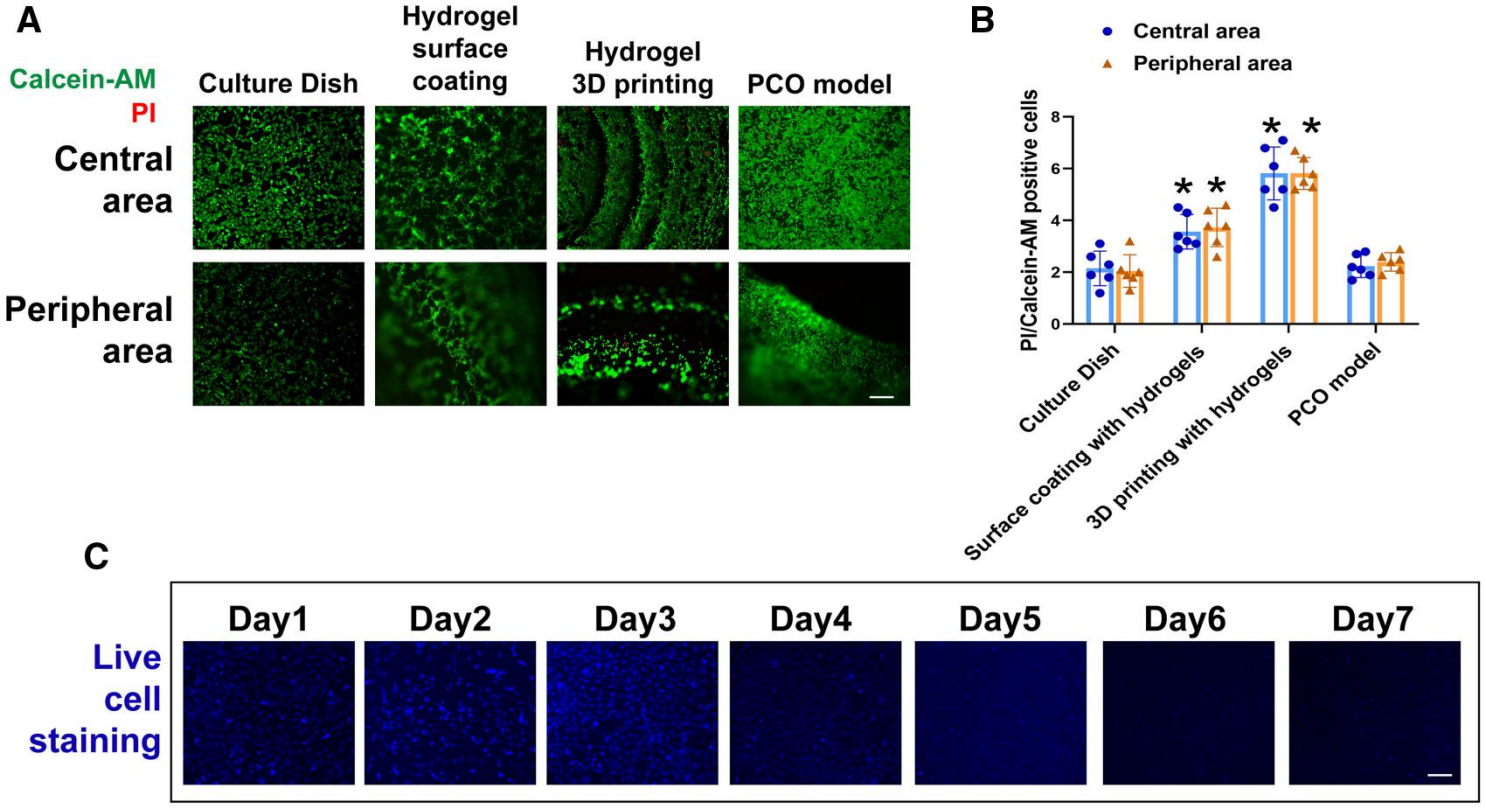
Evaluation of biomaterial compatibility of PCO model. (**A**) Apoptotic cells were analyzed using calcein-AM and PI double staining, where calcein-AM positive represents live cells and PI positive represents dead or dying cells. Scale bar= 50 μm. (**B**) The ratio of PI-positive cells (apoptotic cells) to calcein-AM-positive cells (live cells) among the different groups at the central and peripheral areas. Statistical significance was denoted as *, *P* < 0.05 (*n* = 6). (**C**) Representative immunofluorescence staining shows the staining of live cells in GelMA 60 5%+ PLMA 2% hydrogel from days 1 to 7. Scale bars: 50 μm.

Rhodamine staining intensity served as an indicator of mitochondrial membrane potential (ΔΨm), while DAPI was utilized for nuclear staining. Compared to the control group, both the GelMA30 and GelMA90 groups exhibited a significant decrease in ΔΨm levels. Notably, the ΔΨm levels of the GelMA30 5%+PLMA 2% composite gel were comparable to those of the pure GelMA30 5% gel, while the GelMA90 5%+PLMA 2% composite gel displayed similar ΔΨm levels to the pure GelMA90 5% gel. Conversely, the ΔΨm levels of the GelMA 60 5%, GelMA 60 10%, and GelMA 60 5%+PLMA 2% groups did not exhibit any significant difference compared to the control group. In the GelMA30 and GelMA90 groups, there was no significant difference in ΔΨm levels with increasing concentration. However, with an increase in GelMA 60 concentration, the ΔΨm levels decreased. The GelMA 60 5% + PLMA 2% group demonstrated the highest ΔΨm levels among all the groups studied (Figure 1E and F). Regarding nuclear staining, no significant changes were observed among the different gel groups, indicating that the nuclear structure remained intact irrespective of the gel composition and concentration. Furthermore, HLECs were seeded in a 96-well plate coated with GelMA 60 5%+PLMA 2% for cell tracing. The fluorescence intensity of the cells peaked on the third day and significantly decreased on the sixth and seventh days (Figure 3C). Therefore, GelMA 60 5%+PLMA 2% was chosen as the optimal mixture due to its superior biocompatibility and viability (Figure 1D–F).

We compared various methods for coating the PCO model with hydrogel to better replicate the ultrathin structure of the lens capsule and create optimal conditions for HLEC culture. Calcein-AM/PI staining of HLECs was conducted under different coating conditions. In the absence of any surface coating, only a minimal number of viable cells adhered to the gel model surface, accompanied by a significant presence of apoptotic cells. In contrast, when utilizing GelMA 60 5%, GelMA 60 5%+PLMA 2%, GelMA 90 5%, or GelMA 90 5%+PLMA 2% for 3D printing, HLECs displayed uneven distribution, fusion, deformation and a notable increase in apoptotic cells on the gel model surface, indicating suboptimal growth conditions. Additionally, higher levels of lipid peroxidation were detected (Supplementary Figure S1). Using hydrogel directly for the entire PCO model led to its collapse due to its soft material properties, making precise cell seeding in specific locations such as the equatorial region challenging. Moreover, the hydrogel had a short shelf life, with the printed model dissolving within 1 week, rendering it unsuitable for maintaining the original PCO model structure.

To address these issues, we refined the hydrogel coating method by applying a thin layer directly onto the rigid base of the resin-based 3D-printed structure. Initially, manual coating with hydrogel using a cotton swab proved challenging to control in terms of thickness and uniformity. Consequently, there was no significant increase in the number of HLECs growing on the model surface compared to the uncoated PCO model, and apoptotic cells persisted. We further improved the approach by employing spin-coating to ensure a uniform and ultrathin hydrogel coating on the surface. Various spin-coating parameters were tested, revealing that higher speeds resulted in thinner gel coatings. As the spinning speed increased from 1500 to 2500 rpm, the number of viable cells adhering to the PCO model surface gradually increased. At 2000 and 2500 rpm, the cell distribution became more uniform; however, at 2500 rpm, there was a significant increase in apoptotic cells. Among the different coating methods tested, applying two layers of hydrogel coating at 2000 rpm yielded the best growth status and minimal lipid peroxidation (Figure 2B and C and Supplementary Figure S1). Therefore, this approach was selected as the preferred method for preparing the PCO model.

GelMA hydrogels have been widely applied due to their tunable mechanical properties, excellent photocrosslinking ability and good biocompatibility [39–41]. GelMA is a dual-bond-modified gelatin that can be crosslinked into a gel through UV crosslinking or visible light irradiation upon activation by an initiator. The resulting scaffold structure possesses the characteristics of both natural biomaterials and synthetic biomaterials [40]. As a hydrophilic polymer with a favorable three-dimensional structure, biocompatibility and low immunogenicity, GelMA offers advantages such as injectability, rapid gelation, enhanced mechanical properties and suitability for bioprinting. It promotes cell adhesion and proliferation, making it an excellent platform for cell loading [39, 40]. GelMA can serve as an alternative to artificial substrates or other natural collagen-based hydrogels. Additionally, it contains matrix metalloproteinase sequences that facilitate enzyme degradation, playing a crucial role in tissue repair and wound healing [42, 43]. For example, the utilization of GelMA-based bioactive hydrogel bone scaffolds with osteoconductivity can be combined with bioceramics and bioglass to promote osteogenic mineralization. Additionally, the application of cell-loaded biomimetic scaffolds enables the simulation of the osteogenic microenvironment, thereby facilitating multiple bone defect repair functions [41]. Researchers have also investigated the application of Conductive GelMA/alginate/polypyrrole/graphene hydrogel as a potential scaffold for cardiac tissue engineering, demonstrating its simplicity and low-cost fabrication, tunable mechanical properties, optimal electrical conductivity, blood compatibility, and non-cytotoxicity [44]. In the field of tissue engineering in wound healing, the latest advancement involves the application of 3D-bioprinted GelMA hydrogels for tissue repair, particularly focusing on scalability of 3D bioprinting techniques, durability under physiological conditions and the development of advanced bioinks. These advancements are ushering in a transformative era in regenerative medicine and tissue engineering. The future prospects for GelMA hydrogels include overcoming challenges through multidisciplinary collaboration, advancing manufacturing techniques and embracing personalized medicine paradigms [45].

To ensure aseptic conditions for cell culture in the PCO model, imparting antimicrobial activity to the surface coating of the PCO model is a crucial requirement for successful cultivation of HLECs. Antimicrobial components, when covalently cross-linked into a hydrogel binder, can exhibit long-lasting antimicrobial effects until complete degradation of the hydrogel [46, 47]. ε-Poly-l-lysine (ε-PL) is a short-chain cationic antimicrobial peptide that possesses broad-spectrum antimicrobial activity, excellent biocompatibility and cost-effectiveness. By binding and disrupting microbial membranes, ε-PL induces physiological damage to the microbial population [48]. Photocrosslinked methacrylamide-modified ε-PL (ε-PLMA) is prepared by grafting methacrylic acid (MA) onto the amino group of ε-PL, resulting in a hydrogel with inherent antimicrobial activity [49, 50]. In this study, we developed a stable hybrid hydrogel coating (GelMA60 5% combined with ε-PLMA 2%) on the surface of the PCO model. This hydrogel coating exhibits excellent biocompatibility, stability, antimicrobial activity and a low occurrence of oxidative stress, making it an ideal material for providing a conducive growth environment for HLECs.

### Evaluation of biomaterial compatibility and lipid peroxidation in the PCO model

Compared to cell culture dishes with or without manual hydrogel coating, as well as 3D printing with hydrogels for the complete model, the highest cell count and no significant increase in apoptotic cell numbers were observed in the central and peripheral regions of the PCO model (Figure 3A and B).

**Figure 4.**
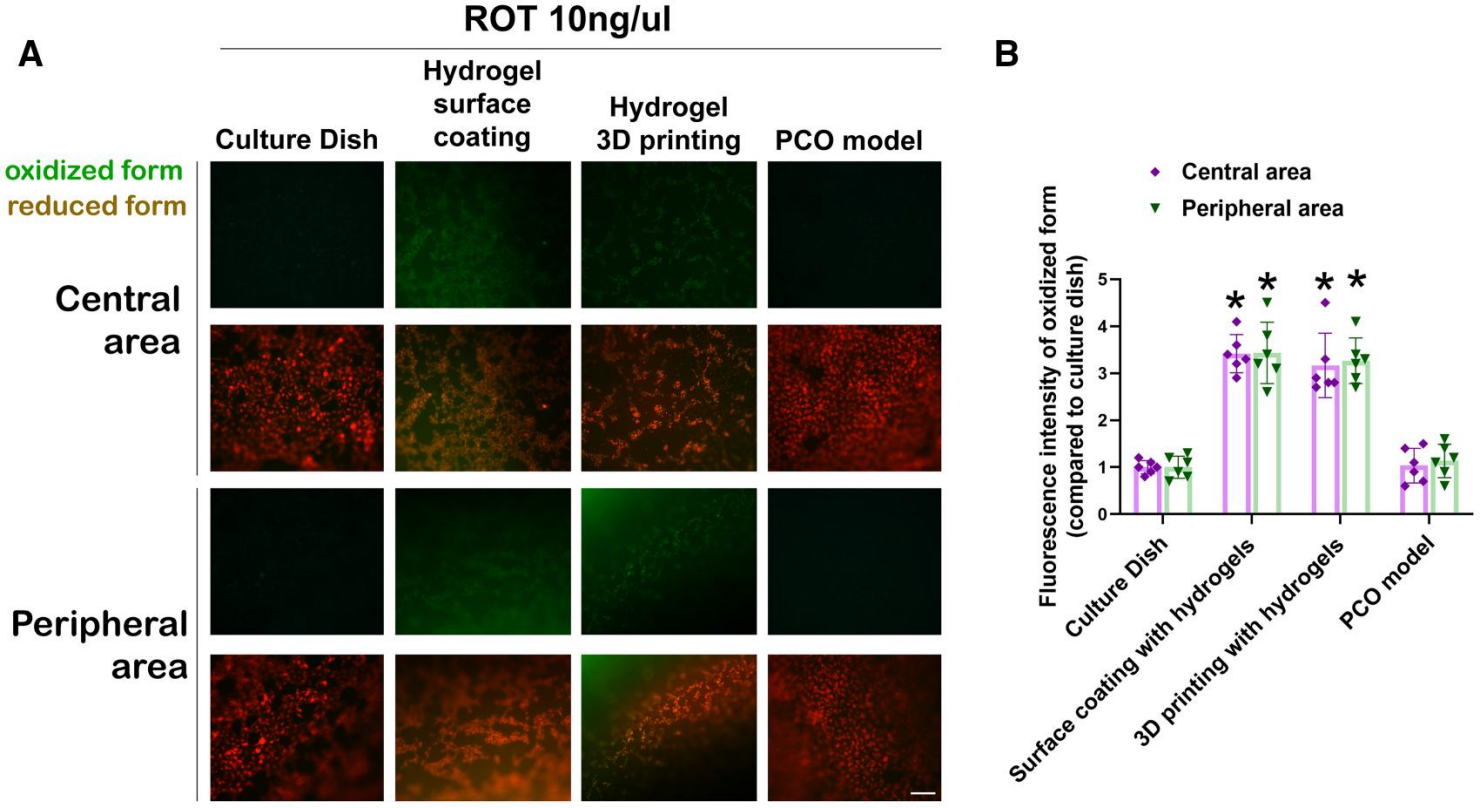
Evaluation of oxidative stress levels of PCO model. (**A**) C11 BODIPY staining reflected the level of oxidative-reduction reactions. Upon oxidation-reduction reactions, the emission wavelength shifted from 591 nm (reduced form) to 510 nm (oxidized form). scale bar: 50 μm. (**B**) Fluorescence intensity of the oxidized form compared to the control group. Statistical significance was denoted as *, *P* < 0.05 (*n* = 6).

C11 BODIPY staining was employed to assess the extent of lipid peroxidation in HLECs under different surface coating methods following 24 h of rotenone-induced oxidative damage. Consistent with viability staining outcomes, the use of GelMA 60 5%, GelMA 60 5%+PLMA 2%, GelMA 90 5% or GelMA 90 5%+PLMA 2% for the complete 3D printing of the PCO model resulted in elevated levels of lipid peroxidation in HLECs cultured within the gel structure. In comparison with cell culture dishes featuring a manual hydrogel coating or those without any coating, the PCO model exhibited more uniform cell adherence and demonstrated significantly lower levels of lipid peroxidation **(**Figure 4**)**.

With the advancement of modern tissue engineering techniques, research on GelMA-based hybrid hydrogel scaffolds holds significant value in various tissue engineering scaffold studies, encompassing important aspects of materials science and biology [26, 29]. However, GelMA as a matrix hydrogel still has some limitations, such as inadequate mechanical strength and the potential impact of UV radiation during photocrosslinking on loaded cells. Therefore, it is crucial to improve the mechanical properties, degradation behavior, microstructure, bioactive functionality and long-term biocompatibility of the scaffold [49, 50].

This study indicates that the direct use of GelMA or a composite hydrogel of GelMA and PLMA for 3D printing of the entire model leads to collapse due to insufficient mechanical strength. Additionally, it fails to accurately replicate the ultrathin structure of the lens capsule, thereby impeding the achievement of ideal biocompatibility and appropriate biomechanical properties necessary for promoting cell adhesion, proliferation and migration. Hence, the implementation of resin-based 3D printing for the rigid base, coupled with a surface hydrogel coating to simulate the ultrathin structure of the lens capsule, becomes crucial. This approach ensures a balance between mechanical support, morphological stability and biocompatibility characteristics.

In this study, the successful implementation of a spin-coating technique for hydrogel coating of the PCO model surface was demonstrated, providing an optimal environment for HLEC culture while preserving the integrity of the model structure. Therefore, a two-layer ultrathin hydrogel coating at 2000 rpm was selected as the preferred method for preparing the PCO model in conjunction with the resin-printed base and lid.

### PCO model for drug evaluation

**Figure 5.**
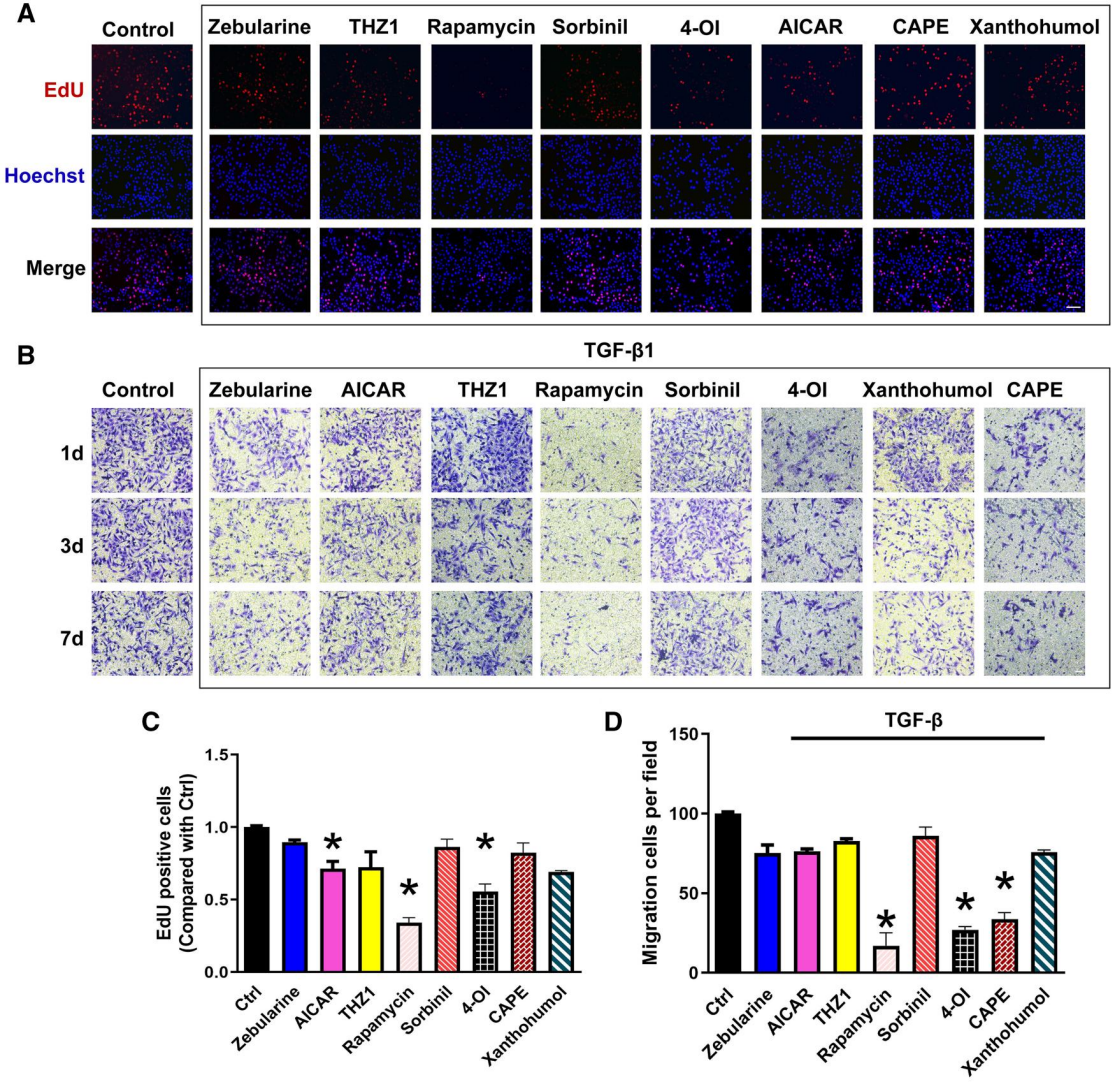
Cell migration after drug treatment in PCO model. (**A** and **C**) HLECs proliferation was assessed by calculating the percentage of EdU-positive cells. Scale bar: 50 μm. *P < 0.05. (**B** and **D**) HLECs migration was analyzed using the transwell assay, under the condition of TGF-β1, following intervention with the eight drugs. Scale bar: 50 μm. *P < 0.05.

**Figure 6.**
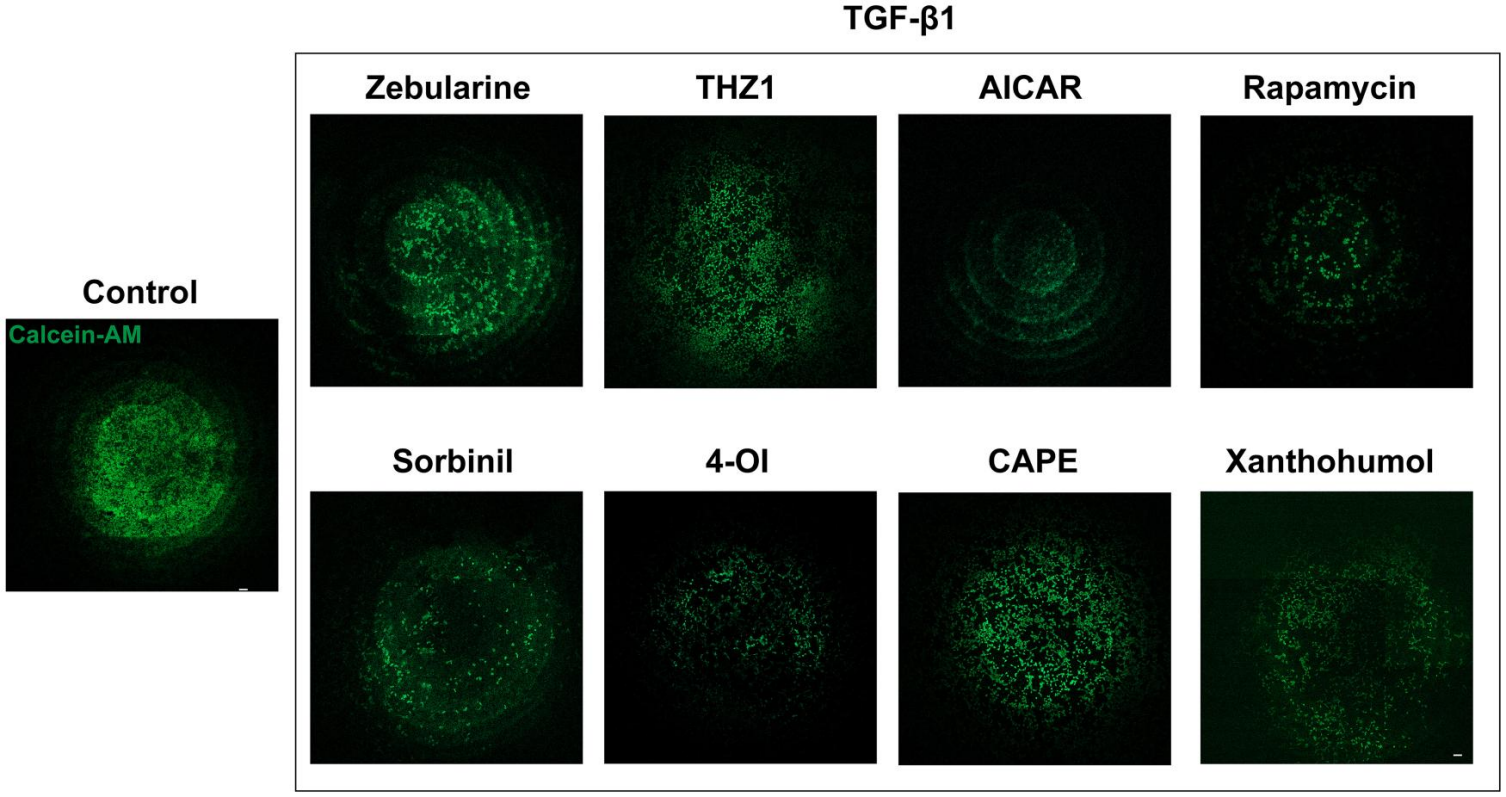
Cell migration evaluation in PCO model after drug treatment. Calcein-AM staining was employed to visualize the migration of live cells toward the central region. of the PCO model after drug intervention. Confocal imaging revealed the final state of cell migration toward the central region after drug intervention for 5 days. Scale bar: 50 μm.

This study applied the PCO model to investigate the potential value of drug intervention. Based on a literature search, eight drugs were selected for their reported antiapoptotic, antioxidative stress, and anti-inflammatory properties: zebularine, AICAR, THZ1, rapamycin, sorbinil, 4-OI, xanthohumol and CAPE. Their effects on HLECs were evaluated to provide theoretical support for PCO drug screening and mechanistic research. Following cell attachment of HLECs in the PCO model, different drugs were added for 48 h of intervention, and EdU staining was used to assess cell proliferation. Compared to the control group, the interventions with AICAR, rapamycin and 4-OI significantly reduced cell proliferation, indicating their inhibitory effects on PCO (Figure 5A and C). After 24 h of TGF-β1-induced proliferation, HLECs were subjected to different drug interventions. Following 1, 3 and 7 days of intervention, the HLECs were digested from the PCO model and seeded into transwell chambers to observe cell migration. Compared to the control group, under TGF-β1-induced proliferation, rapamycin, 4-OI and CAPE exhibited the most significant inhibitory effects on proliferation (Figure 5B and D). After peripheral seeding and cultivation of HLECs in the PCO model for 7 days, confocal microscopy was used to observe cell growth toward the center. Live cell staining by calcein-AM demonstrated that rapamycin and 4-OI exhibited the most significant inhibition of proliferation, followed by AICAR (Figure 6). These results indicate that the PCO model can evaluate the impact of drugs on cellular function through different intervention and observation methods. Among them, AICAR, rapamycin and 4-OI hold potential as therapeutic agents for PCO treatment.

During the development of PCO, the proliferation, migration and EMT of HLECs are considered critical pathological mechanisms [51]. Therefore, inhibiting, clearing or killing HLECs to block the initiation and progression of PCO has become a hot research topic [4, 52]. The effects of drugs used to treat PCO can be categorized into several classes: proliferation inhibitors, inflammation inhibitors (immunosuppressants), apoptosis inducers, cell-clearing or killing agents, migration inhibitors, and adhesion inhibitors. These drugs exert their effects by inhibiting inflammatory responses, EMT, and apoptosis-related pathways. Various studies have evaluated the intervention of PCO through local administration or preparation as coatings on IOLs [3, 4, 6, 13, 53]. Previous studies have reported on the *in vitro* experiments with zebularine, THZ1, rapamycin and sorbinil on HLECs and the *in vivo* experiments on PCO prevention, although these have not yet translated into clinical applications [26, 28, 54, 55]. There is currently no PCO-related research available for the other four drugs. In this study, using the PCO model, we assessed the inhibitory effects of different drugs on PCO under conditions of TGF-β1-induced proliferation. Based on a comprehensive evaluation of various experimental results, AICAR, rapamycin and 4-OI exhibited the strongest inhibitory effects on PCO.

Rapamycin, also known as sirolimus, is a novel immunosuppressant that has been proven to inhibit the proliferation, migration and matrix formation of HLECs in both *in vivo* and *in vitro* models, thereby suppressing the occurrence and development of PCO. It has also been used for the development of surface coatings for IOLs to prevent PCO [55–58]. Rapamycin inhibits mTOR by binding to immunophilin FK protein 12 (FKBP-12). Additionally, it can also inhibit IL-2, IL-4 and IL-15 through Ca2+-dependent or Ca2+-independent pathways, thereby interrupting the inflammatory cascade reaction that leads to T-cell activation and proliferation. It plays an important role in inflammation, rejection reactions, angiogenesis and proliferative diseases [54, 55, 58]. Additionally, sustained-release drug formulations based on rapamycin and nanocarrier-loaded sustained-release IOLs have been developed, showing promise for clinical use [29, 55, 59]. This study also confirmed that rapamycin significantly inhibits the proliferation and migration of HLECs in the PCO model with low cytotoxicity, making it an ideal target for drug development.

AICAR is an AMPK activator that interacts with AMPK-activated metabolic pathways in various tissues and cells. It promotes catabolic metabolism and inhibits synthetic metabolic processes when the AMP/ATP ratio is high. Additionally, AICAR can exert metabolic regulatory effects through non-AMPK-dependent pathways [60]. In various cell types, it inhibits cell proliferation through both AMPK-dependent and independent pathways. In the AMPK-dependent pathway, it inhibits protein synthesis by downregulating the mTOR signaling pathway. In the AMPK-independent pathway, it induces imbalances in the NTP and dNTP pools, suppressing cell proliferation. This process is p53-dependent [60–62]. It also exhibits anti-inflammatory properties, reduces cellular fibrosis and ROS damage, and promotes autophagy and mitochondrial function [63].

4-OI is a cell-permeable derivative of itaconic acid, a metabolite of the tricarboxylic acid cycle generated through Immunoresponsive gene 1-catalyzed cis-aconitate. It plays essential roles in metabolism and immunity, regulating inflammation, apoptosis and autophagy. Disrupting the balance of the immune response and tolerance regulated by the ocular microenvironment is closely related to the incidence of PCO [64]. 4-OI can inhibit inflammatory reactions and proliferation by modulating the Nrf2 signaling pathway and the PI3K/AKT/mTOR signaling pathway [64, 65]. The observed inhibitory effects of AICAR and 4-OI on PCO may be mediated through these pathways. AICAR, rapamycin and 4-OI are promising candidates for drug development.

### Global imaging of the PCO model

**Figure 7.**
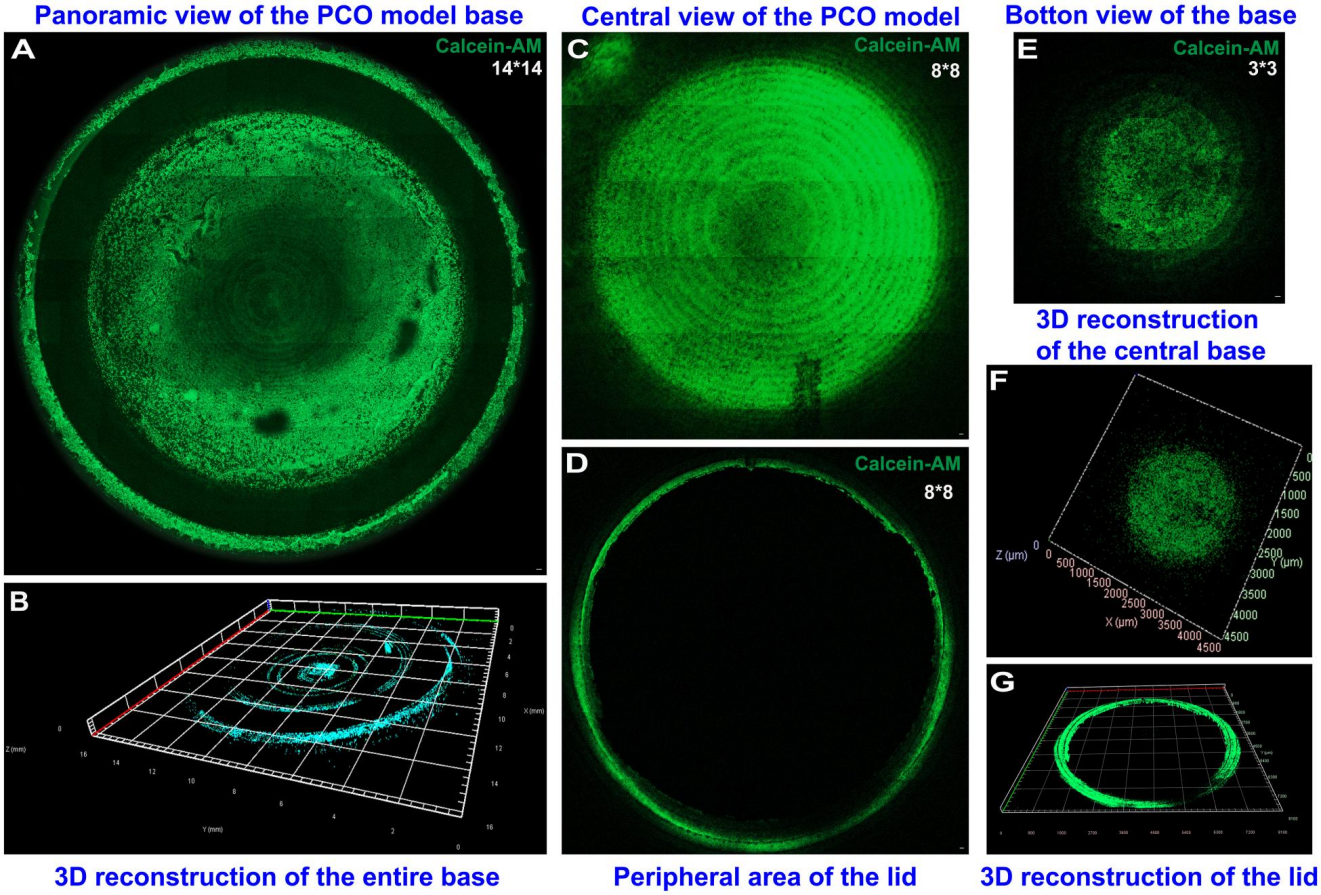
Confocal microscopy imaging of the PCO model. (**A** and **B**) Confocal imaging of the entire PCO model base after calcein-AM live cell staining, showing a 14 × 14 field of view and 3D reconstruction. Scale bar: 100 μm. (**C**) Confocal imaging of the central region of the PCO model, covering an 8 × 8 field of view. Scale bar: 100 μm. (**E** and **F**) Confocal imaging of the central region of the PCO model base, covering a 3 × 3 field of view, and 3D reconstruction. Scale bar: 100 μm. (**D** and **G**) Confocal imaging of the PCO model cover, showing a 8 × 8 field of view and 3D reconstruction. Scale bar: 100 μm.

To obtain optimal panoramic imaging of the PCO and observe the distribution of cells, we utilized confocal microscopy to scan and capture images of the entire base of the PCO model. The height of the PCO model base, designed in this study, falls within the range of the focal length of the 5× objective lens of the confocal microscope, allowing for panoramic imaging to better visualize the growth of HLECs in the PCO model. For imaging, a 14 × 14 field of view was used to capture the overall range of the PCO model and perform 3D reconstruction (Figure 7A and B). Additionally, an 8 × 8 field of view was employed to capture the central region of the base (Figure 7C), while a 3 × 3 field of view was utilized to capture detailed images of the core area of the base (Figure 7E). Subsequent 3D reconstructions were performed (Figure 7F). Furthermore, imaging of the PCO model cover was conducted using an 8 × 8 field of view to capture the distribution of HLECs around the cover, followed by 3D imaging (Figure 7D and G). The results demonstrated that HLECs exhibited robust growth throughout the PCO model, with an even distribution, preserved cell integrity, and the absence of clustering, deformation or collapse. 3D imaging revealed that the spatial distribution of HLECs in the PCO model accurately simulated the structure of the lens capsule, providing a robust scaffold.

Previous studies on PCO models have mainly relied on macroscopic observations through overall photographs and microscopic observations of localized cell enlargement. However, there is no literature reporting how to observe the entire PCO model under a microscope, particularly obtaining comprehensive confocal imaging and observation. To observe the growth of HLECs in the entire PCO model and perform microscopic structural analysis and three-dimensional imaging, this study designed a spiral structure with an open-close mechanism specifically tailored to the dynamic range of confocal microscopy. By opening the top cover, key regions of the PCO model were exposed, allowing for observation, photography and 3D assessment of the entire PCO structure after live cell staining. The results demonstrated that the cell growth status in this PCO model was excellent, establishing an ideal observation protocol.

## Conclusion

In conclusion, this study successfully developed a novel PCO model that accurately replicates the anatomical structure of the human lens. The meticulously designed parameters of the model simulate the postcataract surgery state of the lens capsule, while the 3D-printed hollow base provides stability and support. The application of an ultrathin coating of GelMA and PLMA composite hydrogel creates an optimal microenvironment for HLEC growth, closely resembling the composition and structure of the lens capsule. The threaded structure allows for easy manipulation and observation, facilitating precise seeding of cells and drug interventions. Additionally, confocal microscopy enables comprehensive panoramic imaging of the model. With excellent reproducibility and reusability, this PCO model serves as an invaluable tool for research and offers substantial theoretical support for drug investigations in the field of PCO. The application prospects of the PCO model include the ability to simulate the anatomical structure of the lens *in vitro* for biomimetic cell culture, the construction of a local microenvironment system for lens physiology and the pathogenesis of PCO development, enabling better research into the disease mechanisms of PCO. Additionally, it can be utilized for various drug intervention studies, analyzing the effects of different drugs on intervening in the occurrence and development of PCO, providing an experimental basis for precision intervention.

## Supplementary Material

rbae020_Supplementary_Data
