# Supplementary figures and images for: Fabrication of a 3D bioprinting model for posterior capsule opacification using GelMA and PLMA hydrogel-coated resin
Source: Regen Biomater. 2024 Mar 1;11:rbae020. doi: 10.1093/rb/rbae020 (PMC10963077; doi:10.1093/rb/rbae020)

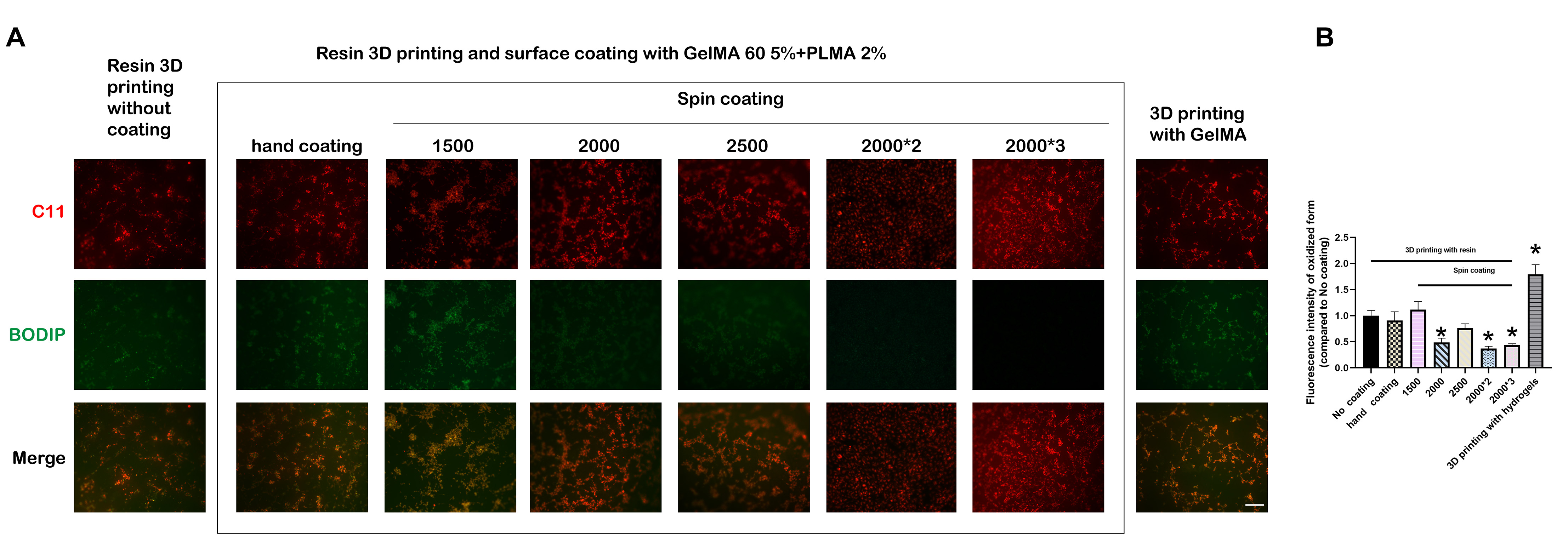

Supplement: rbae020_Supplementary_Data [file rbae020_supplementary_data.zip › Fig S.jpg]
